# Expression of parathyroid hormone-related protein confers malignant potential to mucoepidermoid carcinoma

**DOI:** 10.3892/or.2013.2393

**Published:** 2013-04-08

**Authors:** KYOSUKE NAGAMINE, TETSUYA KITAMURA, AYA YANAGAWA-MATSUDA, YOICHI OHIRO, KANCHU TEI, KYOKO HIDA, FUMIHIRO HIGASHINO, YASUNORI TOTSUKA, MASANOBU SHINDOH

**Affiliations:** 1 Department of Oral Pathology and Biology, Hokkaido University Graduate School of Dental Medicine, N13 W7, Kita-ku, Sapporo 060-8586, Japan; 2 Department of Oral and Maxillofacial Surgery, Hokkaido University Graduate School of Dental Medicine, N13 W7, Kita-ku, Sapporo 060-8586, Japan; 3 Department of Vascular Biology, Hokkaido University Graduate School of Dental Medicine, N13 W7, Kita-ku, Sapporo 060-8586, Japan

**Keywords:** PTHrP, mucoepidermoid carcinoma, malignancy, cancer-associated fibroblast

## Abstract

Parathyroid hormone-related protein (PTHrP) is known to induce bone resorption by activating RANKL as well as PTH. PTHrP plays a central role in humoral hypercalcemia, and its expression has been reported to be closely associated with bone metastasis of breast carcinoma. PTHrP expression in oral squamous carcinoma cell lines was investigated, and PTHrP was expressed in oral squamous cell carcinoma cell lines similar to that in a prostate carcinoma cell line. Mucoepidermoid carcinoma is the most common malignant salivary gland tumor composed of different types of cells including a squamous component. Its clinical behavior is highly variable and ranges from slow-growing and indolent to locally aggressive and highly metastatic. We examined the PTHrP expression in mucoepidermoid carcinoma and assessed the significance of its correlation with clinicopathological features. Immunohistochemical detection of PTHrP was carried out in 21 cases of mucoepidermoid carcinoma in the head and neck region. PTHrP was highly detectable in intermediate and epidermoid cells, and abundant expression of PTHrP in intermediate cells had a significant association with cancer malignancy, including lymph node metastasis and/or tumor recurrence. These results suggest that PTHrP expression can be used as a prognostic factor for mucoepidermoid carcinoma.

## Introduction

Parathyroid hormone-related protein (PTHrP) was originally identified as a major factor responsible for humoral hypercalcemia in malignant tumors such as lung and breast carcinomas (1,2). PTHrP produced by tumors and other cells binds to the common PTH/PTHrP receptor in osteoblasts and activates expression of receptor activator of the NF-κB ligand (RANKL), which promotes the maturation of pre-osteoclasts into osteoclasts which consequently induces bone resorption and hypercalcemia (3,4). PTHrP is produced by certain malignant tumors, and is involved in malignant conversion of breast, colon and prostate cancers by increasing cell proliferation, survival, adhesion, migration and invasion (5–7). We previously reported that PTHrP is highly expressed in oral carcinoma cell lines and that it promotes malignancy of oral cancers (8).

Mucoepidermoid carcinoma is the most common malignant tumor of the major and minor salivary glands, accounting for ~30% of salivary gland malignancies (9,10). Its clinical behavior is highly variable and ranges from slow-growing and indolent to locally aggressive and highly metastatic (11,12). Histologically, mucoepidermoid carcinoma is comprised of 3 different cell types: mucinous cells, intermediate cells and epidermoid cells. Their growth patterns range from cystic to solid to infiltrative. These parameters have been incorporated into several different grading systems that have been correlated with prognosis and, therefore, play an important role in treatment decisions (9). However, various cases of mucoepidermoid carcinoma with poor prognoses are estimated to have low-grade malignancy upon histological examination. Therefore, we evaluated the PTHrP expression in mucoepidermoid carcinoma and herein discuss its role in malignancy.

## Materials and methods

### Patients and tissue samples

Twenty-one patients who consulted the Department of Oral Surgery, Hokkaido University Hospital, and who were diagnosed as having mucoepidermoid carcinoma were examined. Informed consent was obtained from the patients prior to the samples being used. The experiment was conducted under the ethical guidelines of Hokkaido University Hospital. TNM classification was carried out according to the UICC criteria, and tumors were graded according to the World Health Organization guidelines of 2005.

### Western blotting

Human oral squamous cell carcinoma cell lines HSC2, HSC3 and HSC4 [Japanese Collection of Research Bioresources (JCRB), Osaka, Japan) were used in the present study. PC-3, a prostate carcinoma cell line, was used as a positive control. The cells were maintained in Dulbecco’s modified Eagle’s medium (DMEM) supplemented with 10% fetal bovine serum (FBS). The cells were lysed in lysis buffer [10 mM Tris-HCl (pH 7.4), 5 mM EDTA, 150 mM NaCl, 10% glycerol, 1% Triton X-100, 0.1% SDS and protease inhibitor cocktail] (Roche Diagnostics, Indianapolis, IN, USA) for 20 min on ice and clarified by microcentrifugation. The supernatant was subjected to SDS-PAGE and transferred to polyvinylidene difluoride membranes (Bio-Rad Laboratories, Hercules, CA, USA). A PTHrP rabbit polyclonal antibody (Y201; Yanaihara Institute Inc., Shizuoka, Japan) was used for detection of PTHrP in the cultured oral squamous cell carcinoma cell lines.

### Immunohistochemical analysis

Immunohistochemical detection of PTHrP, α smooth muscle actin (αSMA) and CD34 was conducted using paraffin-embedded tissue sections. Sections (5-μm) were deparaffinized and rehydrated. They were immersed in 3% hydrogen peroxide in distilled water for 5 min to block endogenous peroxidase activity followed by 1% BSA in phosphate-buffered saline (PBS) for 10 min. They were then exposed to the primary rabbit polyclonal antibody for PTHrP, αSMA and CD34 for 1 h at room temperature. After washing with PBS three times, Simple Stain MAX PO (Nichirei Biosciences, Tokyo, Japan) was used for 30 min, and sections were visualized with EnVision Plus kits/HRP (Dako, Tokyo, Japan) at room temperature. The peroxidase reaction products were developed with 3,3′-diaminobenzidine, and the sections were counterstained with hematoxylin.

The PTHrP expression ratio of mucoepidermoid carcinoma was calculated by counting the number of positive tumor cells over the total number of tumor cells at a magnification of ×400 in three different areas. The PTHrP expression ratio of mucoepidermoid carcinoma was measured using Nanozoomer with NDP view software (Hamamatsu Photonics, Hamamatsu, Japan) and expressed as: PTHrP expression of mucoepidermoid carcinoma (%) × area of each type of cell.

### Statistical analysis

Data concerning the PTHrP expression ratio of mucoepidermoid carcinoma were analyzed and compared with the two-sample t-test for differences in means. The criterion for statistical significance was P<0.05.

## Results

### Clinical features of the cases examined

The clinical features of the cases are shown in Table I. There were 12 male and 9 female patients. The mean age of the patients was 56 years (range, 23–75 years). There were a wide variety of primary sites. The primary sites of the tumors were the parotid gland in 4 patients, submandibular gland in 1, sublingual gland in 1 and minor salivary glands in the other 15 cases. TNM classification was performed according to the guidelines of the International Union Against Cancer TNM classification system. Patients were followed up for 5 years with regard to the prognosis, lymph node metastasis and/or tumor recurrence. The TNM classification of tumor size was T1 in 5 (24%) patients, T2 in 10 (47%), T3 in 4 (19%) and T4 in 2 (10%) patients. Nodal status was N0 in 17 patients, N1 in 1 and N2 in 3 patients. Five patients presented with subsequent metastases including regional lymph nodes or distant organs and tumor recurrence during the 5-year follow-up period.

### PTHrP protein is expressed in oral squamous cell carcinoma cell lines

**Figure 1:**
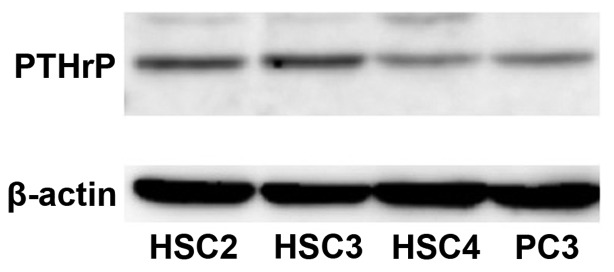
Western blot analysis of PTHrP expression. Cells were lysed in lysis buffer and subjected to western blotting using a rabbit polyclonal antibody for PTHrP. Human oral squamous cell carcinoma cell lines, HSC2, HSC3 and HSC4, expressed PTHrP protein at levels that were almost equal to or higher than the level in PC-3, a prostate carcinoma cell line used as a positive control.

To address the role of PTHrP in oral epithelial tumors, we first examined, using western blotting, whether PTHrP was expressed in oral carcinoma cell lines, HSC2, HSC3, HSC4. All of the cell lines expressed PTHrP protein at levels that were almost equal to or higher than the level in PC-3, the positive control prostate carcinoma cell line (Fig. 1).

### PTHrP expression in mucoepidermoid carcinomas is related to cancer metastasis and recurrence

**Figure 2:**
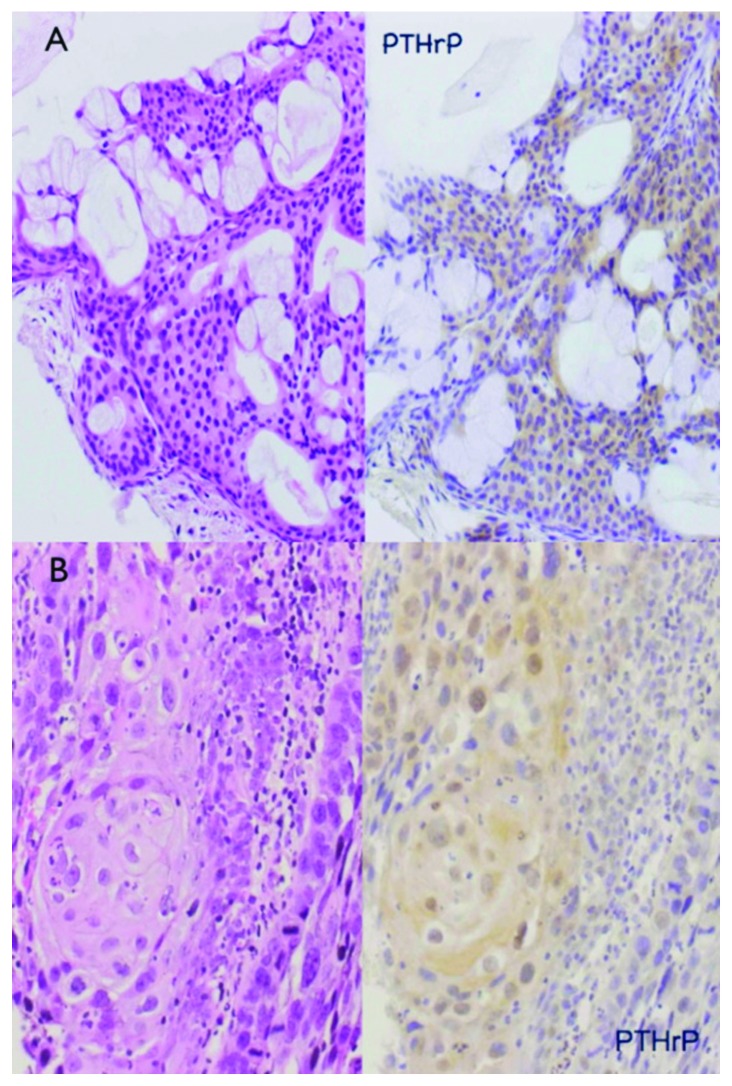
PTHrP expression in mucoepidermoid carcinoma. Immunohistochemical detection of PTHrP was performed in formalin-fixed paraffin-embedded sections of the mucoepidermoid carcinoma cases. PTHrP was predominantly detected in (A) intermediate cells and (B) epidermoid cells. Mucous-producing cells show few positive signals.

**Figure 3:**
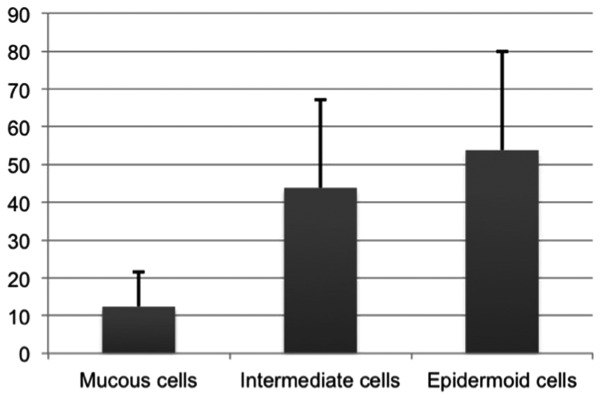
PTHrP-positive cell percentages in the 3 different types of mucoepidermoid carcinoma cells. PTHrP-positive cells were counted in 5 different areas of carcinoma specimens, and the positive cell number/total cell number of each cell type was estimated. The percentage of PTHrP-positive cells was significantly higher in intermediate cells and epidermoid cells.

**Figure 4:**
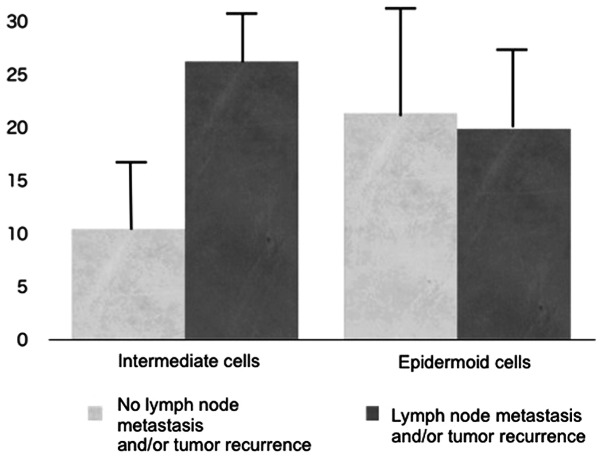
Relationship between PTHrP expression ratio and cancer malignancy. The percentage of the area occupied by each type of cancer cells was measured using a densitometer. The PTHrP expression ratio in each specimen was evaluated by multiplication of the percentage of the occupied area by the PTHrP-positive percentage for each cell type. There was no significant correlation bewteen the PTHrP expression ratio in epidermoid cells and tumor malignancy; however, a significant correlation was observed between the PTHrP expression ratio in intermediate cells and tumor metastasis and/or recurrence.

Immunohistochemical detection of PTHrP was performed for 21 cases of mucoepidermoid carcinoma. Cytoplasmic PTHrP-positive signals were observed in 17 of the 21 cases, and the remaining 4 were PTHrP-negative. PTHrP-positive signals were noted predominantly in intermediate cells (Fig. 2A) and epidermoid cells (Fig. 2B); PTHrP-positive signals were present in 12% of mucous-producing cells, 43% of intermediate cells and 53% of epidermoid cells on average (Fig. 3). Thus, the PTHrP-positive cell percentages were significantly higher in intermediate cells and epidermoid cells. Subsequently, the relationship between the PTHrP expression ratio of intermediate and epidermoid cells in mucoepidermoid carcinoma and cancer behavior was investigated. There was no significant relationship between the PTHrP expression ratio in epidermoid cells and tumor malignancy; however, a significant correlation was observed between the PTHrP expression ratio in intermediate cells and 7 cases of primary and subsequent tumor metastasis and/or recurrence (Fig. 4).

**Figure 5:**
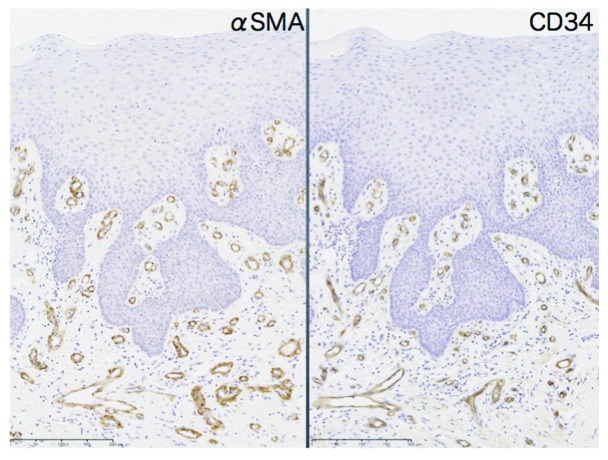
Cancer-associated fibroblast (CAF) distribution in normal mucosa. Immunohistochemical detection of αSMA and CD34 was performed in serial sections of normal oral mucosa. The distribution of αSMA expression was almost equal to that of CD34-positive vascular tissue, and no obvious induction of CAFs was observed.

**Figure 6:**
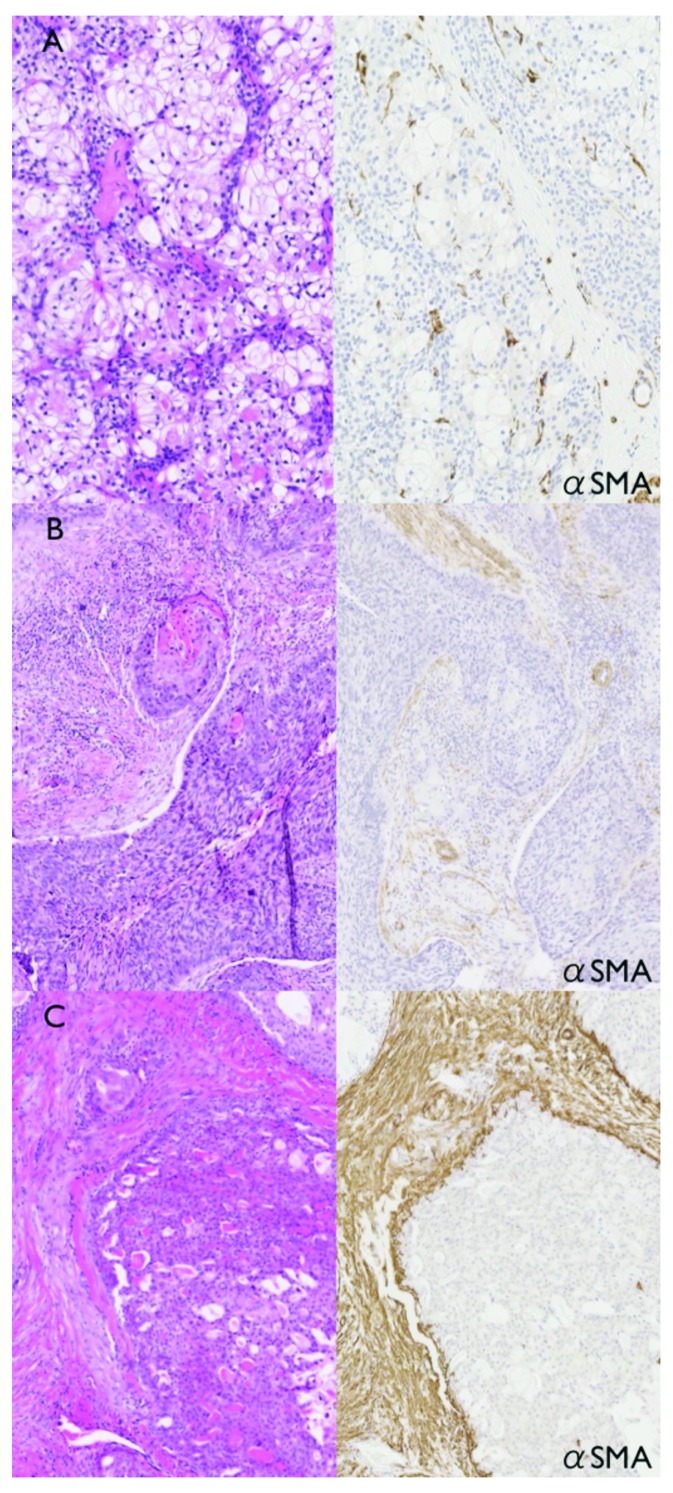
CAF induction in mucoepidermoid carcinoma. αSMA immunohistochemistry was carried out in the mucoepidermoid carcinoma specimens. There were few αSMA-positive fibroblasts around the mucous-producing cancer cells (A), whereas, αSMA-positive CAFs were abundant in the stroma of epidermoid cancer cells (B) and intense αSMA-positive CAFs were widely observed in stromal tissue around intermediate cancer cells (C).

**Figure 7:**
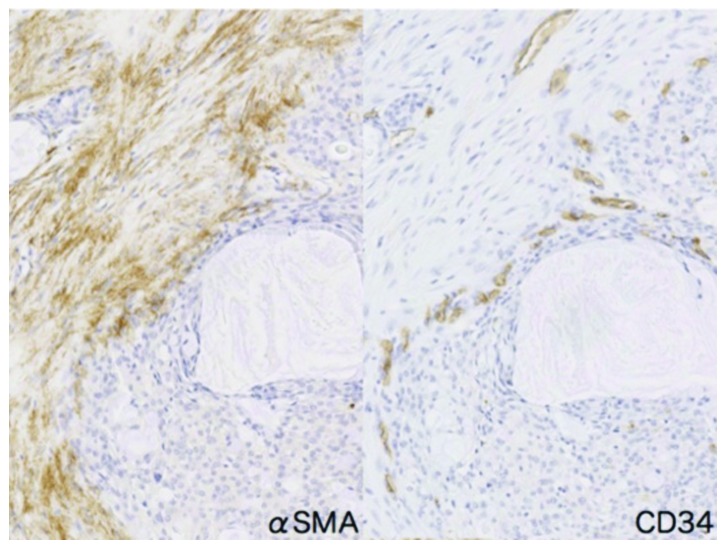
CAFs and proliferating vessels. αSMA and CD34 immunohistochemistry in serial mucoepidermoid carcinoma specimens reveals that CD34-positive small vessels exist adjacent to αSMA-positive CAFs in mucoepidermoid carcinoma.

Cancer-associated fibroblast (CAF) induction was estimated by αSMA expression in cancer stromal tissue. In normal mucosa, αSMA expression was almost equal to the distribution of CD34-positive vascular tissue and no obvious induction of CAFs was observed (Fig. 5). In contrast, αSMA-positive CAFs were induced in stromal tissue of mucoepidermoid carcinoma. Slight induction of CAFs was observed around mucous-producing cancer cells (Fig. 6A). CAFs were more abundant in stromal tissue around epidermoid cancer cells (Fig. 6B) and intense αSMA-positive CAFs were widely observed in stromal tissue around intermediate cancer cells (Fig. 6C). In addition, numerous CD34-positive small vessels were present adjacent to αSMA-positive CAFs in mucoepidermoid carcinoma (Fig. 7).

## Discussion

Mucoepidermoid carcinoma was formerly classified as a benign tumor termed the mucoepidermoid tumor. Most patients have a favorable outcome, yet some patients succumb to the disease. Therefore, mucoepidermoid carcinoma was reclassified as a malignant tumor in the 1992 WHO classification because of its capacity for metastasis regardless of the macroscopic and histologic appearance. Mucoepidermoid carcinoma is categorized as a low- or high-grade malignancy with respect to local recurrence and metastatic ability (13). These criteria were followed until the 2005 classification (9). A grading system with low- to high-grade malignancy using five histopathological features is now utilized. It can be reproducible in defining the prognosis of mucoepidermoid carcinoma patients; however, there are some exceptions concerning tumor grading and prognoses. Thus, it is necessary to establish new methods that can estimate the exact potential for tumor malignancy.

PTHrP was purified from a human lung cancer cell line, and was shown to have biological activities similar to parathyroid hormone (PTH). PTHrP is correlated with the humoral hypercalcemia of malignancy (2). Clinical evidence supports another important role for PTHrP in malignancy as a mediator of the bone destruction associated with osteolytic metastasis (7,14). PTHrP expression by breast carcinoma cells may provide a selective growth advantage to bone due to its ability to stimulate osteoclastic bone resorption (1). Furthermore, growth factors such as transforming growth factor-β (TGF-β), which are abundant in bone matrix, are released and activated by osteoclastic bone resorption and may enhance PTHrP expression and tumor cell growth (14,15). Moreover, PTHrP overexpression was found to increase mitogenesis and decrease apoptosis in a human breast cancer cell line. Clones of MCF-7 cells that overexpress wild-type PTHrP show significantly higher laminin adhesion and migration. This indicates that PTHrP may play a role in breast cancer metastasis by upregulating proinvasive integrin expression, and controlling PTHrP production in breast cancer may provide a therapeutic benefit (7).

In the present study, PTHrP was detected in oral squamous cell carcinoma cell lines, and this raised the possibility that PTHrP may be involved in oral malignancies. Mucoepidermoid carcinoma is composed of mucous-producing, epidermoid (squamoid) cells and those of the intermediate type. We hypothesized that epidermoid (squamoid) cells show a higher level of PTHrP expression, and we found that PTHrP expression was predominantly observed both in epidermoid and intermediate cells. Few signals were observed in mucous-producing cells in the mucoepidermoid carcinoma cases. PTHrP-expressing cell volumes were measured by morphometry, and in the cancer cases where intermediate cells abundantly expressed PTHrP there was a significant association with malignancy, including lymph node metastasis and/or tumor recurrence during the 5-year follow-up period. These results indicate that PTHrP is actually expressed in mucoepidermoid carcinoma, and PTHrP expression in intermediate cells is closely related to malignancy.

Recently, the microenvironment surrounding tumor cells has attracted much attention. Stromal cells have been thought to be composed of normal cells; however, according to Hida and colleagues, tumor endothelial cells have abnormalities and different phenotypes compared to normal endothelial cells (16–18). Fibroblasts in cancer stromal tissue were also shown to have different phenotypes and have been termed ‘cancer-associated fibroblasts (CAFs) (19,20). CAFs have been shown to produce TGF-β, which induces epithelial-mesenchymal transition (EMT) (21). We hypothesised that PTHrP affects the extracellular matrix and plays a role in changing the extracellular matrix to CAFs. These cells were induced in stromal tissue in the mucoepidermoid carcinoma cases, and were most often present around intermediate cells of the mucoepidermoid carcinoma. The precise mechanism of development of mucoepidermoid carcinoma remains obscure. However, intermediate cells are thought to be less differentiated than the other cell types, and they are consequently the source of the other cell types in mucoepidermoid carcinoma (22). Our results indicate that PTHrP-expressing mucoepidermoid carcinoma induces CAFs in stromal tissue, in particular around intermediate cancer cells. This may account for the malignant potential of intermediate cells, and suggests that PTHrP expression is a prognostic factor for mucoepidermoid carcinoma.

## Figures and Tables

**Table I:** Clinical features of the mucoepidermoid carcinoma cases examined.

Case no.	Gender	Age (years)	Primary site	cTNM	Subsequent metastasis/recurrence
1	Female	66	Parotid gland	T2N0M0	
2	Male	41	Palate	T2N0M0	
3	Female	35	Buccal mucosa	T2N0M0	Metastasis
4	Female	47	Buccal mucosa	T3N0M0	
5	Female	71	Buccal mucosa	T1N0M0	
6	Male	71	Parotid gland	T3N0M0	
7	Male	64	Sublingual gland	T2N0M0	Metastasis
8	Male	63	Submandibular gland	T3N2bM0	
9	Male	74	Tongue	T1N0M0	
10	Male	60	Alveolar part of mandible	T2N0M0	
11	Male	61	Floor of the mouth	T2N0M0	Metastasis
12	Male	54	Floor of the mouth	T4N2cM0	
13	Male	75	Palate	T2N0M0	Metastasis
14	Female	23	Parotid gland	T2N0M0	
15	Male	54	Floor of the mouth	T4N1M0	Recurrence
16	Male	41	Tongue	T1N0M0	
17	Female	47	Parotid gland	T2N0M0	
18	Female	53	Alveolar part of mandible	T2N0M0	
19	Female	48	Lip	TT1N0M0	
20	Male	69	Palate	T1N0M0	
21	Female	64	Alveolar part of mandible	T3N2cM0	
